# Feasibility and acceptability of a contextualized physical activity and diet intervention for the control of hypertension in adults from a rural subdistrict: a study protocol (HYPHEN)

**DOI:** 10.1186/s40814-024-01456-w

**Published:** 2024-02-03

**Authors:** Kganetso Sekome, Francesc Xavier Gómez-Olivé, Lauren B. Sherar, Dale W. Esliger, Hellen Myezwa

**Affiliations:** 1 Department of Physiotherapy, Faculty of Health Sciences, School of Therapeutic Sciences, University of the Witwatersrand, Johannesburg, 2193 South Africa; 2 MRC/Wits Rural Public Health and Health Transitions Research Unit (Agincourt), Faculty of Health Sciences, School of Public Health, University of the Witwatersrand, Johannesburg, South Africa; 3 School of Sport, Exercise and Health Sciences, Loughborough University, Loughborough, UK; 4 Faculty of Health Sciences, School of Therapeutic Science, University of the Witwatersrand, Johannesburg, South Africa

**Keywords:** High blood pressure, Nutrition, Rural, South Africa

## Abstract

**Introduction:**

In rural and remote South Africa, most strokes and ischaemic heart diseases are as a consequence of hypertension, which is a modifiable risk factor. The widely recommended therapeutic approaches to control hypertension are through physical activity and diet modifications. However, there is a lack of culturally sensitive community-based, lifestyle interventions to control hypertension among rural African adult populations. We designed an intervention which recommends adjusting daily routine physical activity and dietary behaviour of adults with hypertension. This study aims to evaluate the feasibility and acceptability of HYPHEN in a rural community setting.

**Methods:**

We aim to recruit 30 adult participants with a self-report hypertension diagnosis. A one-arm, prospective design will be used to assess the feasibility and acceptability of recruitment, uptake, engagement, and completion of the 10-week intervention. Recruitment rates will be assessed at week 0. Intervention uptake, engagement, and adherence to the intervention will be assessed weekly via telephone. Blood pressure, body mass index, waist-hip ratio, urinary sodium, accelerometer-measured physical activity, and 24-h diet recall will be assessed at baseline and at 10 weeks. Qualitative semi-structured interviews will be conducted at 10 weeks to explore feasibility and acceptability.

**Discussion:**

This study offers a person-centred, sociocultural approach to hypertension control through adaptations to physical activity and dietary intake. This study will determine whether HYPHEN is feasible and acceptable and will inform changes to the protocol/focus that could be tested in a full trial.

**Trial registration number:**

PACTR202306662753321.

## Introduction

The World Health Organization projects that in the African region, by 2030, noncommunicable diseases (NCDs) will be the biggest cause of death [1]. The relative burden of NCDs is expected to increase as both HIV-/AIDS-related mortality declines with the roll-out of antiretroviral therapy and the region continues to experience the rapid urbanization of communities [2]. Hypertension, stroke, and ischaemic heart disease are among the most common NCDs causing premature deaths among adults living in South Africa [3]. Over half of strokes and ischaemic heart disease in South Africa are caused by hypertension, and these cardiovascular and cerebrovascular diseases are reported to be more prevalent in rural areas compared to urban areas [3].

The high prevalence of hypertension in South Africa will likely result in increased mortality and morbidity in rural compared to urban areas due, in part, to poorly organized health care systems, intermittent drug supply, and shortage of health care workers [4–6]. Reduced salt and caloric intake and increase in physical activity are the widely recommended therapeutic approaches to meeting national hypertension targets [7–9].

There is a paucity of research that provides a profile of daily physical activity practices of rural South African adults living with hypertension. A study by Oyeyemi and colleagues [10] assessed physical activity for rural South Africans using short versions of physical activity subjective assessment which does not provide information about the domain of physical activity (transportation, job, leisure, and housework) performed each day. Another study using a short version physical activity questionnaire by Smart Mabweazara [11] in the general population concluded that adults in rural Western Cape engaged in more physical activity than urban adults from the same province. A further study by Tomaz and colleagues [12] assessed physical activity for middle-aged and older rural adults and recommended that physical activity interventions should be targeted for adults from the age of 40 years in order to provide health benefits earlier in life.

Available literature on dietary practices of rural South African adults has provided successful intervention recommendations for hypertension, but the recommendations are not sustained in the long run. For example, a study by Charlton and colleagues [13] provided recommendations of food replacement which participants could not afford due to high unemployment rates in rural South Africa and the fact that recommended food was not part of the participants’ daily diet [14]. Another study by Najam and colleagues [15] reported that adults in rural South Africa have good nutrition knowledge, but this did not translate into regular healthy nutrition practices. The literature on physical activity and diet practices for rural South African adults points to a need to have better understanding of the sociocultural influences so that interventions targeting the control of hypertension can consider the realities of this population.

To the authors’ knowledge, there are no interventions to test whether adaptations in physical activity and diet focusing on a contextualized structured health promotion approach could lower high blood pressure in a rural South African adult population with hypertension [16, 17]. There is a need for studies to investigate the feasibility of implementing such interventions. A contextualized intervention is one that is adapted to the local context of the target population and informed by the population needs. Many adults in rural South Africa are involved in daily manual labour as part of their activities of daily living, but this is performed at low intensity and is not well documented [10]. Common manual labour activities reported by adults living in rural South Africa include walking for wood collection, different aspects of farming, yardwork, walking as a means of transport, and housework. These activities are not adequate to paint a full picture of the daily routine practices of rural adults and need further exploration [16, 18]. There is lack of information regarding the type, frequency, and intensity of physical activities performed by rural South African adults. Moreover, many diet-based interventions are usually not affordable due to high costs or not accepted by the rural adults due lack of availability and contextual considerations, hence the need for tailor-made interventions that consider the contextual realities of the population [19, 20].

We followed a systematic process to design an evidence and theory-informed contextualized 10-week physical activity and diet intervention which recommends adjusting routine physical activity and diet practices for the control of hypertension using the intervention mapping (IM) protocol [21] — HYPHEN (*HY*pertension control using *PH*ysical activity and di*E*t in a rural co*N*text). Intervention mapping is used as a planning approach to develop theory- and evidence-based health promoting intervention programmes. Intervention mapping has been suggested as a useful and effective tool to further improve the development and application of theories to promote nutrition and physical activity [22]. The tasks in the IM protocol are iterative with the programme planner revisiting previous steps throughout the process so that all objectives, outcomes, and determinants are addressed. This process allows for the development and delivery of the intervention to be replicated.

### Aim and objectives

The aim of this study is to implement and evaluate the feasibility and acceptability of a contextually appropriate educational programme for hypertension control in adults from a rural South African population.

The primary objectives of the study are as follows:

- To present the social and cultural beliefs, perceptions, and practices regarding physical activity and diet which influenced the development of an intervention for the control of hypertension in adults living in rural South Africa
- To implement a contextually appropriate educational intervention programme for the control of hypertension using physical activity and diet
- To determine feasibility of the intervention through assessing recruitment, retention, and attrition rates, intervention uptake, engagement, adherence to the intervention, completion, and the evaluation measures
- To determine acceptability of the intervention through individual interviews
- To assess fidelity of intervention delivery using a checklist centred around the behaviour change techniques

The secondary objective of the study is as follows:

- To present descriptive statistics data on health-related indicators (blood pressure, physical activity, diet consumption, body mass index, urine sodium, and waist-hip ratio) before and after the intervention.

## Methods

### Study design

This feasibility study will be a one-arm community-based, prospective study. Feasibility and acceptability of recruitment, the intervention (HYPHEN), and associated outcome measures will be assessed using a mixed-method approach.

### Study site and population

This study will be conducted at Bushbuckridge subdistrict in Mpumalanga province where the Agincourt Health and Demographic Surveillance System (HDSS) has been running since 1992. The Agincourt HDSS covers an area of 450 km^2^ with 116,000 individuals (approximately 52,000 older than 18 years) living in approximately 20,000 households distributed in 31 villages [23–25]. We will sample adults aged 40 years and above within the Agincourt HDSS from the Health and Aging in Africa: A Longitudinal Study of an INDEPTH (International Network for the Demographic Evaluation of Populations and Their Health) community in South Africa (HAALSI) [26]. The prevalence of hypertension in this subdistrict for people aged 40 years and above is 57%, defined as a self-reported diagnosis of hypertension (systolic blood pressure ≥ 140 mmHg or diastolic blood pressure ≥ 90 mmHg) or self-reported taking medications for hypertension control [26].

### Sample size

A study which provides guidance on conducting feasibility studies by Tickle-Degnen [27] states that feasibility studies are not expected to have large sample sizes as they do not need to power statistical null hypothesis testing. However, Arian and colleagues [28] in their review of current best practice state that the sample size of feasibility studies should be adequate to estimate critical parameters such as recruitment rate. An overall sample size of 30 has been recommended by Lancaster, Dodd, and Williamson [29]. We will aim to recruit 30 participants at the beginning of the intervention.

### Patient identification, consent process, and ethical considerations

Eligible participants will be invited telephonically via the Agincourt HDSS from a cohort of adults with hypertension. The details of the study will be explained through a telephone call, and if the adult demonstrates interest, a household visit will be conducted to provide further information, answer any questions, and obtain written informed consent to participate. The study has received the necessary ethical clearance from the University of the Witwatersrand Human Research Ethics Committee (clearance number: M 210282) and the local Provincial Department of Health Research and Ethics Committee (clearance number: MP_202106_001). We do not anticipate any adverse events to occur because of participating in this intervention; therefore, no data monitoring committee will be required. Participants already taking antihypertensive medications will continue this treatment as usual.

#### Inclusion criteria

- Ages 40 years and older
- Self-reported diagnosis of hypertension or self-reported taking medications for hypertension control
- Lived in the district study setting for minimum 6 months prior to start of intervention.

#### Exclusion criteria

- Dependent on wheelchairs for mobility

### Preparation procedures

The primary author will train three research assistants on the entire protocol of the study including questionnaire administration, anthropometric measurements, and education of the participants. The summary of the study schedule is outlined in Table 1.

**Table 1:** COM-B component and behaviour change techniques (BCT) used in the structured group education session targeting hypertension

Model component	Taxonomy category	BCT	Definition	Finding from needs assessment	Example
Capability	Shaping knowledge	Information about antecedents	Provide information about antecedents (e.g. social and environmental situations and events, emotions, cognitions) that reliably predict performance of the behaviour	Lack of knowledge about blood pressure and normal blood pressure values Negative influence from community resulting in myths about causes of hypertension	Knowledge about what causes hypertension and normal blood pressure values Knowledge on ways to control blood pressure such as reducing salt intake and increasing physical activity Awareness of social triggers/barriers such as cooking for the family Awareness and correction regarding common myths

### Intervention

The intervention will comprise of three components: a structured group education session, individualized physical activity education, and individualized dietary education over a period of 10 weeks (Tables 1, 2 and 3). The duration of the intervention was informed by recommendations from similar studies [30, 31]. The three components of the intervention were informed by a needs assessment from unpublished data. The first part of the needs assessment established the profile of physical activity levels of hypertensive adults in the rural subdistrict. Results found that males reported more vigorous physical activity than females at work. Females reported high frequency of moderate-intensity physical activity inside and outside the house. The second part explored the sociocultural perceptions about physical activity and dietary practices which influence behaviour change for the control of hypertension in adults from the rural subdistrict. Some key findings included a lack of variety in the daily consumed diet, and the usual diet was mainly influenced by affordability and availability. Physical activity practices were mainly influenced by community perceptions of age- and gender-related roles.

**Table 2:** COM-B component and behaviour change techniques (BCTs) targeting individual’s physical activity behaviour

Model component	Taxonomy category	BCT	Definition	Findings from needs assessment	Example
Capability	Shaping knowledge	Instruction on how to perform a behaviour (identified by the participant) Goal setting (behaviour)	Advise or agree on how to perform the behaviour (includes ‘skills training’) Set or agree a goal defined in terms of the behaviour to be achieved	Most daily activities are performed at low intensities or in sitting positions	Changing positions (from sitting) when performing activities Increasing pace of usual active daily tasks Avoid sedentary behaviour Identifying a list of usual daily physical activities performed
Opportunity	Shaping knowledge	Instruction on how to perform a behaviour (identified by the participant)	Advise or agree on how to perform the behaviour (includes ‘skills training’)	Some daily activities performed were of moderate to high intensities	Emphasize the importance and benefits of moderate to high intensity physical activities on hypertension control
Motivation	Feedback and monitoring	Self-monitoring of behaviour	Establish a method for the person to monitor and record their behaviour(s) as part of a behaviour change strategy	Lack of motivation to maintain behaviour change recommendations	Participant to tick which activity(ies) they achieved on a weekly basis

**Table 3:** COM-B component and behaviour change techniques (BCTs) targeting individual’s dietary behaviour change

Model component	Taxonomy category	BCT	Definition	Findings from needs assessment	Example
Capability	Shaping knowledge Antecedents	Instruction on how to perform a behaviour Avoidance/reducing exposure to cues for the behaviour Goal setting	Advise or agree on how to perform the behaviour (includes ‘Skills training’) Advise on how to avoid exposure to specific social and contextual/physical cues for the behaviour, including changing daily or weekly routines Set or agree a goal defined in terms of the behaviour to be achieved	Diet is rich in high salt content such as processed food; use of high salt spices for cooking	Education on high sodium content food items from usual diet Education on reduction in salt consumption and high salt spices Finding alternative options for food with lower sodium content (for example brown bread instead of white bread) Limiting cooking salt to one teaspoon per day
Opportunity	Shaping knowledge	Information about antecedents	Provide information about antecedents (e.g. social and environmental situations and events, emotions, cognitions) that reliably predict performance of the behaviour	Habitual consumption of self-grown vegetables and naturally growing wild herbs such as nkaka, *Aloe*, and *Moringa*)	Encourage to continue consumption of wild herbs and self-grown garden produce
Motivation	Feedback and monitoring	Self-monitoring of behaviour	Establish a method for the person to monitor and record their behaviour(s) as part of a behaviour change strategy	Lack of motivation to maintain behaviour change recommendations	Participants to tick which dietary adjustments they achieved weekly

**Fig. 1:**
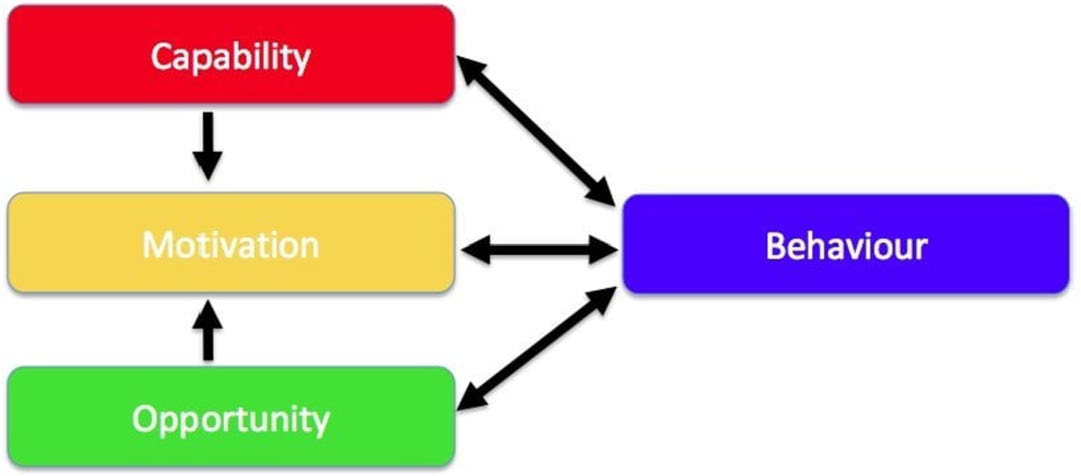
COM-B model of behaviour change [32]

The design of the intervention was underpinned by the COM-B model by Michie, Van Stralen, and West [32] (Fig. 1). The model identifies three factors that need to be present for any behaviour to occur — Capability, Opportunity, and Motivation. Capability is defined as the individual’s psychological and physical capability to engage in the activity concerned. The capability includes having the necessary knowledge and skills. Opportunity is defined as all the factors external to the individual and prompt or make the behaviour possible. Motivation is defined as brain processes that energize and direct behaviour, and it includes habitual processes, emotional responding, and analytical decision-making. Potential influence between components in the model can occur, for example opportunity and capability can influence motivation (Fig. 1).

#### Intervention component one: structured group education

Participants of the intervention will be invited to a common location in small groups (of between 6 and 10). The main focus of the group education will be to enhance capability through shaping knowledge and skills to engage in the proposed intervention. The trained research assistants will deliver the education using a printed educational pamphlet. Some topics to be covered in the discussion (informed by a contextual need assessment) will include knowledge about what causes hypertension and normal blood pressure values, knowledge on ways to control blood pressure such as reducing salt intake and increasing physical activity, awareness of social triggers/barriers such as cooking for the family, awareness, and correction regarding common myths. The group education is expected to last up to 90 min per session.

#### Intervention component two: individualized physical activity education

Participants who attended the group education session will be visited at their home by a trained research assistant to provide individual education on physical activity. Capability will be enhanced by first asking the individual to set goals by identifying the physical activities which they perform as part of their daily routine from a list (informed by a needs assessment). The research assistant will educate the participant on how to adapt performance of the physical activities. This may include changing positions (from sitting) when performing activities, increasing pace of usual active daily tasks, and avoiding sedentary behaviour. Opportunity will be enhanced by emphasizing the importance and benefits of moderate-to-high intensity physical activities on hypertension control. Participants will be motivated by weekly telephone calls where they will be asked to indicate which activities they were able to achieve that week and encouraged where they fall short. The research assistant will keep record of everyone’s weekly performance so that feedback and motivation can be enhanced during weekly telephone feedback.

#### Intervention component three: individualized dietary education

To enhance capability, individual education on how to make dietary changes will be provided on the same visit as the physical activity education. The research assistant will provide education on food items which contains high sodium content from the participant’s usual diet, education on reduction of salt consumption and high salt spices, finding alternative options for food with lower sodium content (for example brown bread instead of white bread), limiting cooking salt to one teaspoon per day, and removing salt from the table during meals. Opportunity for consuming healthier food items will be enhanced by encouraging habitual consumption of self-grown vegetables and naturally growing wild herbs which participants already have access to in their communities. Participants will be motivated through weekly monitoring calls where they will be asked to list which dietary adjustments they achieved weekly. The research assistant will keep record of everyone’s weekly dietary habits so that feedback and motivation can be enhanced during weekly telephone feedback.

The authors are cognizance of the under-pinning theories that influence behaviour change such as the social cognitive theory [33]. The primary variable of interest in this theory of motivation is self-efficacy, which concerns someone’s belief in his/her capabilities to successfully execute necessary courses of action to satisfy situational demands [33, 34]. According to Bandura [34], a high expectation of self-efficacy and outcome expectancy leads to greater adherence to a recommended activity (in our case, physical activity and diet adaptations). Alongside the COM-B model, we developed taxonomies of behaviour change techniques (BCTs) (Tables 1, 2 and 3) to identify active intervention ingredients and allow for future replication [35, 36]. The BCTs are classified using the Behaviour Change Taxonomy [36].

The scope and sequence of the intervention are provided in Table 4. The intervention will run over a period of 10 weeks. During week 0, baseline individual assessments of blood pressure, body mass index, waist-hip ratio, and urine sample collection will be conducted. Participants will be asked to wear a wrist-worn accelerometer for seven consecutive days. They will also be asked to complete a 24-h diet recall questionnaire. During week 1, participants will be invited to a group education session limited to up to 10 participants per group. Participants will be asked to return the accelerometer device during the group session. The research assistants will note down the recruitment rate, intervention uptake, and any reasons for refusal to take up the intervention. Week 2 of the intervention will consist of individual household visits to provide participants with physical activity and diet education based on set goals and to provide printed pamphlet of the education components. The research assistants will telephone the participants weekly for monitoring support and motivation. On week 10, the research assistants will conduct household visits and reassess all baseline measurements. Participants will be asked to wear the acceleromer for 7 days as a post-intervention measure. Adherence rate, completion rate, follow-up rate, and fidelity will be assessed. An exit interview will be conducted to assess intervention acceptability.

**Table 4:** Scope and sequence of the intervention

Programme components	Sequence			
	**Week 0**	**Week 1**	**Week 2 (household visit)**	**Week 10 (household visit)**
**Group educational programme**		Group education sessions		
**Individualized physical activity**			• Goal setting • Physical activity intervention education • Provide printed intervention pamphlet	Final data collection of self-monitoring physical activity tool
**Individualized diet**			• Goal setting • Dietary intervention education • Provide printed intervention pamphlet	Final data collection of self-monitoring physical activity tool
**Anthropometric measurements**	Baseline individual assessment • Demographic data • Blood pressure • BMI^a^ • WHR^a^ • Urine sample collection			Follow-up individual assessment • Blood pressure • BMI^a^ • WHR^a^ • Urine sample collection
**Physical activity assessment**	Baseline accelerometer wear for 7 days	Return accelerometer		Follow up accelerometer wear for 7 days (collect after 7 days)
**Dietary assessment**	Nutrition questionnaire—24-h diet recall			Follow up nutrition questionnaire- 24HR diet recall
**Feasibility parameters**		• Recruitment rate • Intervention uptake and engagement • Reasons for refusal		• Adherence rate • Completion rate • Follow-up rate • Acceptability interview • Fidelity

^a^*WHR* waist-hip ratio

^a^*BMI* body mass index

### Self-monitoring support

Due to reported low literacy levels in the HAALSI cohort [26, 37], self-monitoring support which does not require the participants to read or write will be provided. The trained research assistants will telephone each participant once weekly to ask questions about the participant’s identified physical activity and dietary goals. The research assistant will have a copy of each participant’s printed intervention and record progress. Participants will be asked to provide personal and alternative contact number. Where a participant cannot to be reached via telephone, a household visit will be conducted. The self-monitoring support is also noted as an active behaviour change technique due to its ability to enhance motivation of adhering to an intervention [35].

### Outcomes and criteria for success

In line with the primary objective (c) of the study, the criteria adapted for judging the feasibility of progressing to a larger-scale evaluation (Table 5) were initially developed by Avery and colleagues’ [38] top Ten tips for guiding the decision to progress from a pilot to a definitive trial and later reproduced by Hynes and colleague [39]. The criteria were also discussed by the study authors guided by existing literature.

**Table 5:** HYPHEN progression criteria

HYPHEN progression criteria	Go—proceed to larger scale (RCT)	Amend—proceed with changes	Stop—do not proceed unless changes possible
Feasibility of participant recruitment—can 30 participants be recruited in 1 week?	If 30 participants are recruited in 1 week (100%)	If 15 to 29 participants are recruited in 1 week (50 to < 100%)	If < 15 participants are recruited in 1 week (< 50%)
Feasibility of patient retention—can at least 80% of recruited participants be retained until study end?	≥ 24 (80%) retained	21–24 (70–80%) retained	< 21 (70%) retained
Feasibility of engagement—can at least 80% of recruited participants provide complete weekly data	≥ 24 (80%) engaged	21–24 (70–80%) engaged	< 21 (70%) engaged
Feasibility of intervention implementation	Delivery of intervention judged strongly feasible by qualitative data	Delivery of intervention judged feasible by qualitative data	Delivery of intervention judged possibly feasible by qualitative data

#### Primary feasibility outcome measures

For primary objective a of the study, nine focus-group discussions were conducted as part of the needs analysis process, and the outcome is presented in Tables 1, 2 and 3. For primary objective b of the study, we followed a model of behaviour change as outlined in Fig. 1, and the outcomes are presented in Tables 1, 2 and 3. Primary objective c will be assessed as per Table 5.

The feasibility study parameters which will be used to inform the design of a larger HYPHEN intervention trial are outlined in Table 4. Demographic data (age, sex, employment status, type of employment) of participants who refuse to participate in the intervention will be recorded as well as their reasons for refusal. Acceptability of the intervention will be assessed using an in-depth semi-structured interviews through a self-developed interview guide. All participants who were recruited will be invited for the interviews including those who did not complete the intervention. The interview topics and questions will focus on predetermined themes of participants’ perceived expectations of the intervention, benefits, motivations, and barriers. These topics will be explored in relation to participants age, sex, and employment status. Participants will also be asked to rate their overall satisfaction with the intervention using a Likert scale [40]. Fidelity of intervention delivery will be assessed by the primary author to ascertain whether the field workers provided the intervention as intended using a checklist framed around the intended BCTs (Table 6).

**Table 6:** Fidelity of delivery of intervention

**Definition** [41]	**Intervention BCTs**	**Example**
**Intervention is delivered as intended**	Information about antecedents	Facilitator provided education on all identified aspects of physical activity and diet
	Goal setting	Facilitator identified and made a tick of the participant’s goals for physical activity and diet modifications
	Instruction on how to perform a behaviour	Facilitator explained to the participant how to perform identified goals with images on a printed handout
	Self-monitoring of behaviour	Facilitator followed with each participant weekly and noted with a tick based on participant’s responses
	Avoidance/reducing exposure to cues for the behaviour	Facilitator explained with pictures to the participant how to identify and avoid/replace food items with lower sodium salt

#### Secondary outcome measures

Secondary outcomes which will be assessed are outlined in Table 7 and are as follows: (i) Blood pressure will be measured using a oscillometry blood pressure monitor, (ii) body mass index will be measured using a stadiometer and a portable LED weighing scale, (iii) waist-hip ratio will be measured using a rubber tape measure, (iv) urinary sodium will be measured by collecting participants urine sample, (v) physical activity will be measured using an Axivity AX3 wearable device on the wrist, and (vi) diet will be assessed using a 24-h diet recall questionnaire.

**Table 7:** Secondary outcome measure outline

Variable	Tool used	Manufacturer	Participant position/instruction	Procedure
**Blood pressure**	Oscillometry blood pressure monitor	OMRON M3 comfort, Japan	Sitting on a chair with back supported with feet on the ground. Cuff wrapped on upper arm	Three measurements recorded and the average of the last two will be calculated [42]
**Body mass index**	Portable stadiometer	SECA, Germany	Standing on the scale with shoes off, no cap or hat worn on head	Body mass index will be calculated by dividing the participant’s weight in kilograms by the participant’s height in metres squared [43]
	Portable LED weighing scale	DQUIP, India	Standing on the scale. Shoes off, no heavy clothing worn, no items in pockets	
**Waist-hip ratio**	Rubber tape measure (centimetres)	N/a	Waist: participant standing shoulder width apart, measuring around participant’s waist at the smallest point Hip: participant standing shoulder width apart, measuring around participant’s hip at the widest part	Waist circumference divided by hip circumference [44]
**Urinary sodium**	Urine sample	N/a	N/a	On-the-spot urine sample collected in a 300-ml container and stored at temperatures of − 80 °C in a laboratory upright biomedical freezer. Samples will be analysed in a clinical laboratory which adheres to good clinical laboratory practice (GCLP) standards. Samples will be tested only for sodium levels
**Physical activity**	Axivity AX3	Axivity Ltd., UK	Participant to wear the axivity wristwatch and instructed not to remove it	Device worn on the nondominant wrist with an adjustable watch strap over 7 full days consecutively
**Diet**	24-h diet questionnaire	[45]	Participant asked to recall all food consumed in the past 24 h	Record type of food consumed for breakfast, lunch, dinner, and any snack meals

### Data and statistical analysis

#### Quantitative analysis

A Consolidated Standards of Reporting Trials (CONSORT) flowchart will be used to present the flow of participants throughout the intervention. The CONSORT flowchart will also present reasons for declining participation or withdrawing. Descriptive statistics will be used to present baseline statistics and outcomes of feasibility. Depending on data distribution, mean and standard deviation or median and interquartile range will be described. Pre- and post-intervention outcome change will be assessed using a paired *t*-test if data is evenly distributed. If data is unevenly distributed, the Wilcoxon signed-rank test will be used. Confidence interval is set at 95%.

#### Qualitative analysis

The interviews will be audio-recorded, transcribed verbatim, and analysed using Braun and Clarke’s approach to thematic analysis [46]. An independent transcriber will be employed to perform the transcription, the first author will perform the initial codes and themes, and the co-authors will confirm the generated codes and themes. Data will be described narratively. Participants’ direct quotations will be provided and will be used to judge whether the participants perceive the intervention to be acceptable. Positive responses from the participants will indicate acceptability (Table 5).

### Patient and public involvement

In the needs assessment prior to intervention design, stakeholder engagements were undertaken with hypertensive adults exploring their perceptions of sociocultural influences on diet and physical activity. Hypertensive adults were also consulted on their perceived intervention needs and preferred educational approaches before designing the intervention. Local dieticians were consulted about current approaches to hypertension control and learning points from community-based interventions. Community members/the public were also consulted for social issues that are likely to enhance/inhibit success of the intervention. The findings from the various engagements led to the development of the 10-week intervention. The involvement of local research assistants was an important aspect to consider overcoming any trust and cultural issues involved.

## Discussion

In this study, we will seek to implement and then test the feasibility of a community-based intervention for hypertensive adults that incorporate the patient’s perceptions about their social and cultural beliefs regarding physical activity and dietary practices. This study may lead to recommendations that will assist in the control of high blood pressure particularly for adults in a rural South African or similar population. The results from this study may provide a novel contribution to the understanding of contextual factors about physical activity, diet, and implementation of interventions for a rural, South African adult population. Data generated from this study will inform the design and implementation of a larger-scale intervention of HYPHEN, if appropriate.

## Data Availability

Data sharing is not applicable to this article as no datasets were generated or analysed during the current study.

## Abbreviations

NCD: Noncommunicable disease
BCT: Behaviour change technique
CONSORT: Consolidated Standards of Reporting Trials
HDSS: Health and demographic surveillance system
HREC: Human Research Ethics Committee
IM: Intervention mapping
